# easyDAS: Automatic creation of DAS servers

**DOI:** 10.1186/1471-2105-12-23

**Published:** 2011-01-18

**Authors:** Bernat Gel Moreno, Andrew M Jenkinson, Rafael C Jimenez, Xavier Messeguer Peypoch, Henning Hermjakob

**Affiliations:** 1 Software Department, UPC-BarcelonaTech, Barcelona, Spain; 2 European Bioinformatics Institute, Hinxton, Cambridge, UK

## Abstract

**Background:**

The Distributed Annotation System (DAS) has proven to be a successful way to publish and share biological data. Although there are more than 750 active registered servers from around 50 organizations, setting up a DAS server comprises a fair amount of work, making it difficult for many research groups to share their biological annotations. Given the clear advantage that the generalized sharing of relevant biological data is for the research community it would be desirable to facilitate the sharing process.

**Results:**

Here we present easyDAS, a web-based system enabling anyone to publish biological annotations with just some clicks. The system, available at http://www.ebi.ac.uk/panda-srv/easydas is capable of reading different standard data file formats, process the data and create a new publicly available DAS source in a completely automated way. The created sources are hosted on the EBI systems and can take advantage of its high storage capacity and network connection, freeing the data provider from any network management work. easyDAS is an open source project under the GNU LGPL license.

**Conclusions:**

easyDAS is an automated DAS source creation system which can help many researchers in sharing their biological data, potentially increasing the amount of relevant biological data available to the scientific community.

## Background

In recent years the amount of biological data generated have been increasing greatly, and with the advent of new technologies like next generation sequencing this trend is likely to increase. Additionally, new analysis and re-analysis techniques help to produce better and more accurate derived results every day. Making all this data and results publicly available can be of great benefit for the scientific community as a whole, since valid biological data can be used both by field researchers and by those developing new methodologies and algorithms. Sharing raw research data allows others to conduct re-analysis and meta-analysis, usually reinforcing the previous results or even producing novel ones. ArrayExpress [1] and GEO [2] have had a very positive effect on microarray research development and have been heavily used in the development of both new data analysis techniques and biological knowledge. Sharing research results eases rapid spreading of new findings and its incorporation in ongoing research increasing its overall usefulness.

The effects of this sharing can be greatly increased if data and results are made publicly available using some kind of machine readable standard format allowing them to be seamlessly used by other researchers.

While making the raw data available as supplementary material attached to the publication of a paper can be useful and other researchers can certainly use it, its integration with data from other sources will still be difficult and will not help fully automatic approaches such as workflows. However, if this same data is made available in a standard machine readable format it can be easily integrated with data coming from other standard sources and automatically displayed and analyzed.

**Figure 1:**
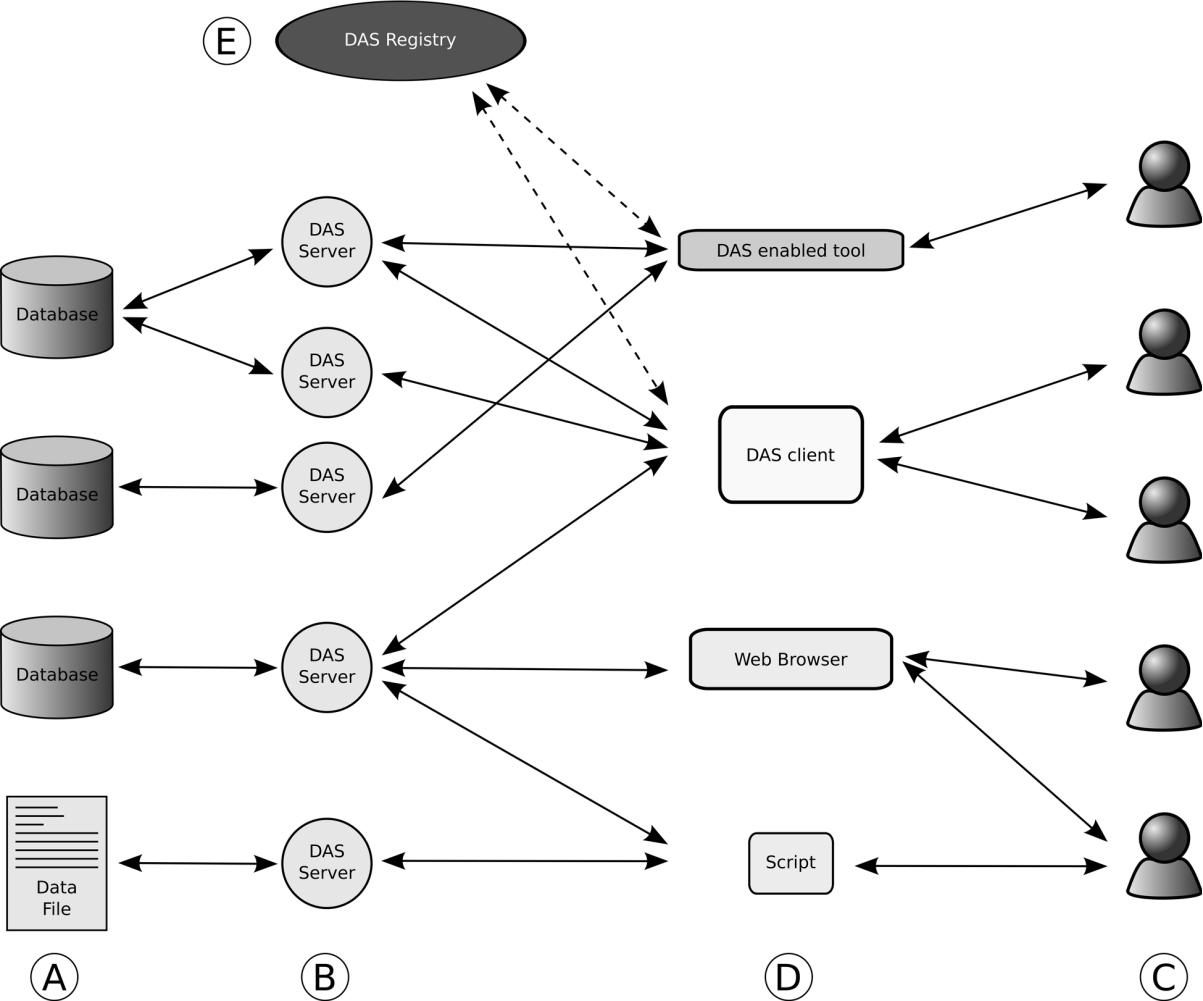
**Overview of DAS**. Data is stored in either databases or data files (A). DAS servers (B) offer a common interface to access that data, the DAS protocol. Users (C), can use different client types (D) including specific DAS clients, internet browsers and non-visual scripts, to access the data. Optionally, clients can access the DAS Registry (E) to retrieve a list of available DAS sources.

The Distributed Annotation System (DAS) is a complete system for sharing annotations on biological sequences. It comprises a standard XML based file format, an accurate definition of the semantics of the data -based on the use of ontologies of biological terms-, and an HTTP based REST style protocol for sharing those annotations [3-5]. Figure 1 is an overview of the DAS system. Many Annotation Servers can provide annotations of sequence objects provided by a Reference Server, for example Ensembl or UniProt. While initially designed to annotate genomic sequences, DAS has support for both genomic and protein annotations, for sequence alignment data, and for structural information. Other federated systems exist for other types of biological data, such as PSICQUIC [6] for molecular interaction data.

DAS client-server architecture was designed around the idea of having a small number of complex clients integrating data coming from, potentially, many of different simple sources. Some examples of DAS clients are Ensembl [7], Dasty2 [8], GBrowse [9], Jalview [10], SPICE [11], PeppeR [12], DASher [13]. Sharing biological data on DAS allows data providers to leverage the DAS ecosystem and make it easy to integrate their data with other existing sources.

DAS server software is available in different programming languages, such as ProServer [14] in Perl and Dazzle [15] and MyDAS [16] in Java. However, despite the idea of DAS servers being simple, setting up a DAS server is not a trivial task. DAS servers allow for a great flexibility on where the actual data is stored and how it is structured. Usually the backend is database, but files and other options are also viable. The downside of this flexibility is that very often data providers will need to implement a custom made data access layer mapping their real data layout to the DAS concepts used in the server and this will have to be done either in Perl or Java. There are many research groups who will not have easy access to people proficient enough in programming to implement that access layer. In addition, setting up and managing an internet accessible machine to host the server can be also difficult or a too big overhead for many data generators, mainly for those with small data sets.

Thus, the challenge: converting all those data generators into data providers, increasing the amount and variety of the biological data available to the scientific community and contributing to the collective annotation of biological sequences.

## Results and Discussion

easyDAS has been developed to help on that conversion offering a web-based and ready-to-use system for biological data sharing using DAS. The user only needs to upload a text file with the data into easyDAS and set a few configuration options, mainly stating what the data represents, and the system will automatically create a new DAS source. Although the DAS source will be automatically created and managed by easyDAS, the user will retain full control over the data and will be able to modify or delete it at any point using the same web interface.

We envision easyDAS being useful for a wide range of potential DAS users. Biologists producing annotations can create DAS sources to take advantage of the DAS infrastructure to integrate their data into Ensembl or other DAS clients. Creating a DAS source can also be used to spread new experimental results and share them with other researchers. Computational biologists and bioinformaticians developing new analysis or prediction tools can easily create new sources with the results of applying those new tools to known datasets making their example runs publicly available and usable. Finally, and since there is no limit on the number of sources to be created by a user, personal datasets can be also published in easyDAS, creating as many temporary sources as needed. This would allow bioinformatics service groups to create tailored sources containing, for example, the set of genes resulting from a microarray analysis or study-specific annotations of proteins. In any case, the user is relieved from the burden of setting up and maintaining their own DAS servers.

easyDAS is fully compliant with the recently approved DAS 1.6 specification [5] and encourages the new systematic semantic annotation of features via an integrated ontology browser based on the Ontology Lookup Service [17].

An easyDAS is freely available at http://www.ebi.ac.uk/panda-srv/easydas.

easyDAS is open source software under the GNU LGPL license. The project is hosted at Google Code and can be freely accessed and downloaded at http://code.google.com/p/easydas/.

### System Description

easyDAS exposes two different entry points to the users. The first one is a web interface where sources can be found, created and managed. The other one is the DAS server, which exposes the data using the DAS protocol. The main components of easyDAS web interface are a users management system, a list of the existing data sources and a wizard for creating new sources.

**Figure 2:**
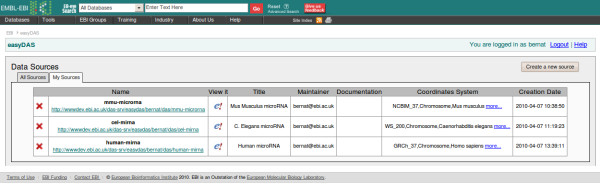
**Sources list**. Screenshot of the main page of easyDAS with the user logged in. The table contains a list of the sources created by the user and offers means to explore and remove them.

The main page in the easyDAS web interface is the list of the available sources with their descriptions and URLs (Figure 2). This list offers easy access to data sources created by any user, facilitating the use and spreading of that data. Links to popular DAS clients like Ensembl are provided too, and will usually attach the source to the viewer automatically. All easyDAS sources can also be registered in the DAS Registry [18] to further increase their visibility.

To create a new DAS source based on a custom dataset, a wizard-like interface will guide the user through the whole process: uploading the file and providing some information about the structure and type of data in the file. This wizard can be invoked clicking on the **"Create a new source" **button in the main page. Although DAS supports alignment and 3 D structure annotations in addition to the standard sequence annotations, those capabilities are currently not heavily used -about 1% of the registered sources offer them- and usually provided by specialized sources. Thus, and since the vast majority of users are willing to share mainly sequence annotations, easyDAS does not support them.

easyDAS provides a rich user interface with standard elements like dialogs and wizards resembling those of a desktop application. It is completely AJAX driven to increase usability and interactivity and to improve system response time.

#### User Registration

Users can register to the system using either OpenID [19] or a traditional username/password pair. All sources created by registered users can be updated, modified or even deleted after creation and can exist for an unlimited time. Sources created by unregistered users will be considered "anonymous" sources and will be online for a limited time only. Anonymous sources cannot be modified or deleted.

When a user is logged into the system, a second tab will be activated in the main page listing only the sources created by the user. The source modification and deletion functionality can be accessed from that table.

#### Source Privacy

It is very important to note that, as almost any other DAS source, easyDAS sources are fully public and accessible by anyone through a simple URL. Although it's possible to mark a source so it's not published in the main easyDAS list, this will not control the access to the actual data. It is not currently possible to create private DAS sources using easyDAS.

#### File Formats

The currently supported file formats are the General Feature Format (GFF) and a versatile implementation of the Comma Separated Values (CSV) where the separator can be any character (although only comma, semicolon and tabulator will be automatically detected). While GFF is a commonly used format that many bioinformatics software can export, CSV can be obtained from any tabular data using a spreadsheet program like Microsoft Excel or OpenOffice Calc.

Some minor extensions and modifications to the formats are supported, like comments on CSV files.

Additionally, easyDAS specific information can be added in the form of special comments containing metadata. Those special comments can be used to pre-configure the source metadata so even less work is required on the web interface. Examples of such comments are the GFF options source-name, source-title or source-maintainer that will be used to pre-populate the corresponding source metadata fields.

#### Semantic Annotation

One of the important additions of the version 1.6 of the DAS specification is the standardization of the semantic annotation of features. Each annotation in DAS has a property describing its type (type), and another describing how it was generated (method). Both properties could already be expressed using ontology terms, but the inclusion of the cvId attribute to specify the controlled vocabulary identifier associated to types and methods and the recommendation of using specific ontologies standardizes those options and makes semantic management of DAS sources and features easier. Specifying ontology terms for feature types will improve data integration and organization in semantic-aware clients like Dasty2 that offer ontology based filtering and grouping. By having most of the sources semantically annotated, easyDAS will be well placed to take advantage of a future semantic search function in the DAS Registry.

**Figure 3:**
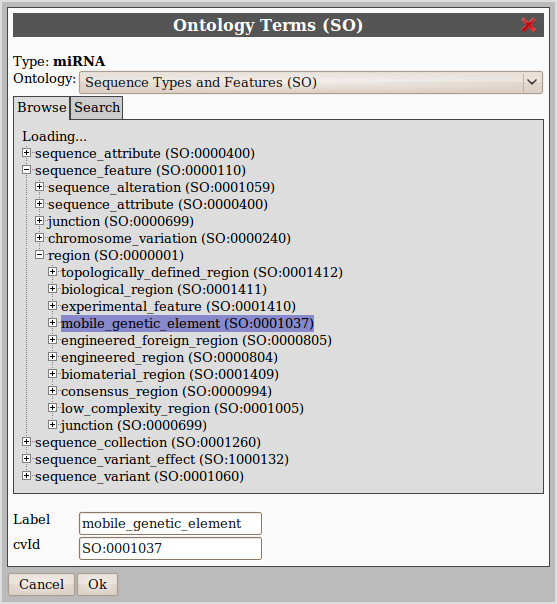
**Ontology browser**. The easyDAS ontology browser widget, used to select ontology terms to semantically annotate the features. It leverages the Ontology Lookup Service webservices to offer both ontology tree browsing and search capabilities.

easyDAS encourages the correct use of those new attributes and has a simple ontology browsing and searching widget (Figure 3). That widget offers interactive browsing of ontologies using on-demand leaf expansion and complete text search capabilities. All that functionality is backed by the web services provided by the Ontology Lookup Service. The ontologies recommended in the DAS specification, Sequence Ontology [20], Biosapiens Ontology [21] and PSI-MOD [22] and Evidence Code Ontology, can be used to annotate the features. Any other ontology available on OLS can be added upon request.

To help in the process of selecting the right ontology terms and to improve annotation consistency, the selected ontology terms are stored in the database and associated to the user and the identifier used in the data file. Thus, when creating other sources using similar data and the same type identifier, the ontology terms will be preselected.

#### Mapping

The mapping of the data file fields into DAS concepts is one of the most important parts in the process of creating a source. The mapping interface, a table showing the data on the file plus a series of comboboxes, is flexible and allows the user to specify the relations as one-to-one, one-to-many or even many-to-many, where it makes sense.

For semantically specified file formats like GFF, a mapping taking that information into account is proposed. For other file formats, when data file fields have names -such as column names on tabular files-some simple pattern-matching heuristics are used to create a proposed mapping. Users can always change that proposal to adapt it to their specific needs.

#### Coordinate Systems

Another important concept in DAS is that of coordinate systems. They uniquely identify the sequences being annotated. In genomic DAS, for example, a coordinate system is defined by a species and assembly -i.e. we can specify that we are annotating the current genomic sequence for human by saying its the assembly "GRCh37" of "Homo sapiens". While not required -there are cases when no suitable coordinate system is available, like when annotating a newly sequenced organism-, it is strongly recommended to specify the coordinate system for a new source. Most of the clients will refuse to integrate sources without a coordinate system, since it's not possible to ensure that the annotations are referring to the same sequence. easyDAS provides a simple coordinate system selector with all the coordinates systems available on the DAS registry. It is possible to filter by any of the fields to help finding the right coordinate system for the source.

### Source Creation

The source creation functionality is based on a wizard-like interface with a series of simple steps that guide the user through the process of defining the new source. At every step as much information as possible is extracted from either the data file or the context to reduce users' work and decisions to the minimum.

The creation process starts with the file Upload form where a simple file selector field is available. While the user can specify the format of the uploaded file, some heuristics are in place to detect the format automatically. This will usually be the best option.

**Figure 4:**
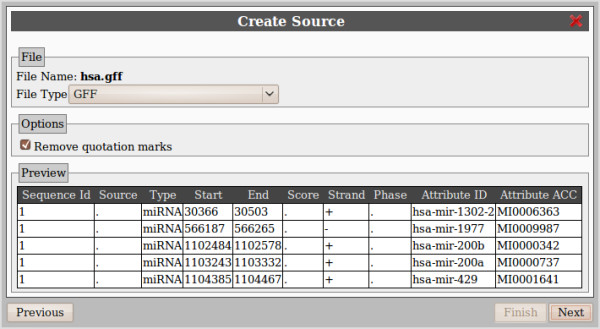
**File format description**. After the data file has been successfully uploaded, this dialog allows to fine tune the file parser. At any moment, a preview of the parsed data is shown.

The second step in the source creation wizard shows a preview of how the file has been identified and parsed and some additional options to further refine the parsing (i.e. removing quotation, defining headers...). In case the automatic format identification mechanism fails, it is possible to amend the format selection in this second step (Figure 4).

The basic source metadata is gathered in the next step. The identifier of the source (which will be part of the source URL), its title, description and maintainer are specified here. The source coordinates system dialog is accessible from this step too.

The fourth step is the mapping form. In this step, the user is required to link the data fields present in the uploaded data file to the standard DAS fields. This is the description of the data required to transform the data in the file into the DAS format. This is the last required step and it is possible to finish the wizard at any point from here.

To help in the simplification of the data files, it is not necessary to specify all the data fields for every feature in the file. The defaults form allows the user to define default values for absent fields or even for partially filled ones. It would be possible, for example, to specify the same method, type or score for all the features at once, maintaining those DAS concepts out of the primary data files.

The last two steps are used to specify the semantic annotations of the features. The ontology browser can be used to select the terms best describing the types and methods of the features in the file.

### Data Storage and Access

The easyDAS interface is available at different address to the web interface and can be queried using standard DAS requests. From a DAS point of view, each registered user has his own DAS server and his sources are created in that server. For example a user with a server name john would have a source called dataset1 available at http://www.ebi.ac.uk/das-srv/easydas/john/das/dataset1 and a list of all his sources at http://www.ebi.ac.uk/das-srv/easydas/john/das/sources. All the DAS requests are served by a slightly customized ProServer [14] also running on the EBI systems.

A MySQL relational database is used to store the sources data and can be accessed by both the web interface and the DAS server.

### Custom easyDAS instances

easyDAS is free open source software. Its source code can be downloaded and modified freely in the terms of the GNU LGPL license. Although the reference instance is running at the EBI, it also means that it is possible to make custom installs of easyDAS independent of the EBI servers. It would be possible, for example, for an organization to provide a custom easyDAS setup in their own network so its groups can publish their data from the organization servers. It would even be possible to install easyDAS inside a private network and setup the included ProServer to reply only to requests coming from inside the network. That could be useful for organizations working with sensitive data but willing to share data between their own groups.

## Conclusions

We have developed a system for the automatic creation of DAS sources. Users can upload data files with sequence annotations in different formats and define and create a new DAS source via a simple web-based wizard. Sources data will be stored on the EBI systems and freely available through a standard DAS interface. Data uploaded to easyDAS can then be easily integrated with other data on DAS using any of the available DAS clients such as Ensembl or Dasty2. easyDAS DAS sources are completely public and no access restrictions of any kind are applied.

As of today, using easyDAS is the easiest and fastest way of sharing a small or medium datasets over the DAS network. We think that this ease will encourage researchers with novel and unavailable datasets to publish and share them increasing the total amount of biological information available to the scientific community. Additionally, easyDAS will help those who need to share biological sequence annotations but can not run their own DAS server.

## Implementation

**Figure 5:**
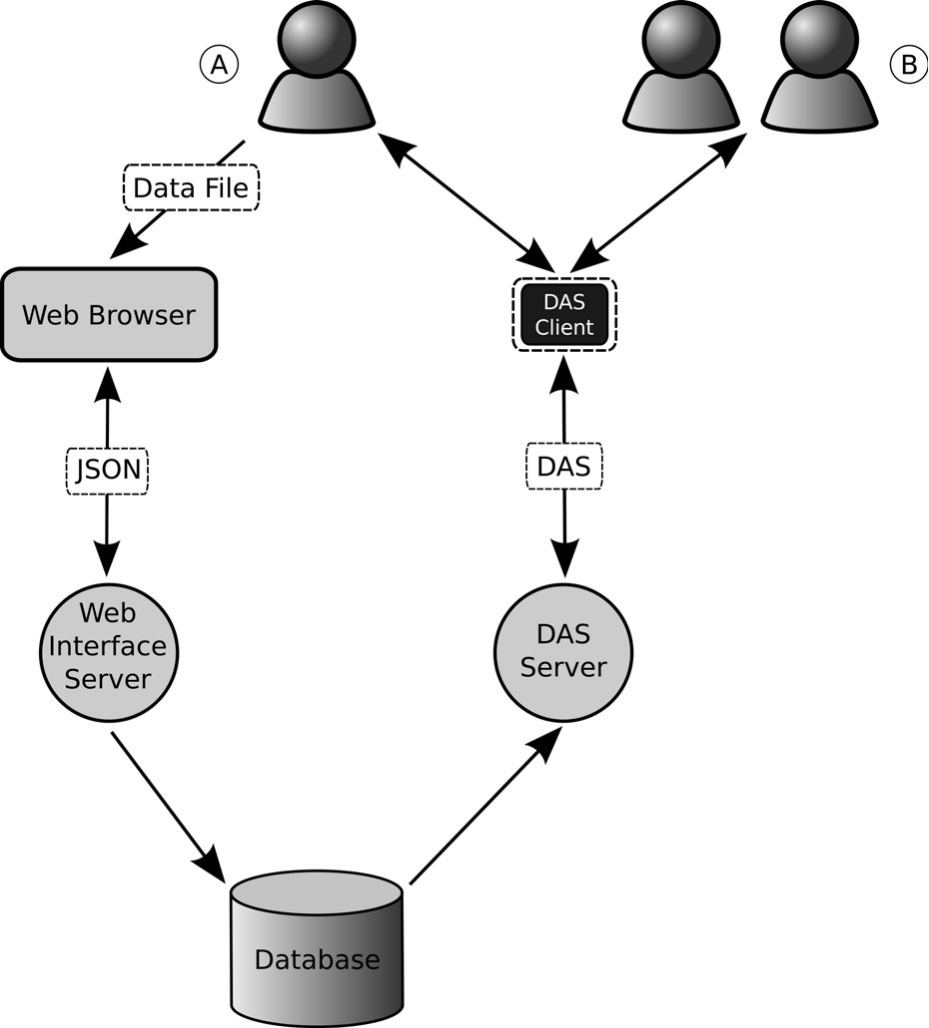
**Implementation overview**. A user (A) can access easyDAS using his web browser and upload a data file. The the easyDAS server, using the users description of the file, will extract the its data and insert it into the database. Anyone (B) can then access that information through the easyDAS DAS server using any of the available DAS clients, which are not part of the easyDAS system.

easyDAS has two different entry points to the system: the web interface and the DAS server. The web interface is a client-server application, with the client being a web application written in Javascript and the server a set of cgi Perl scripts. The web interface is in charge of uploading and parsing the users datasets and offers the interface to define and manage the DAS sources. The other entry point, the DAS server, is a standalone ProServer instance with some minor modifications and is the one responding to the actual DAS commands to access to the data. A MySQL relational database accessible by both the web interface server side and ProServer stores the actual data (Figure 5).

### Web interface

The web interface has two different parts, the client and the server. Communication between those parts is based on AJAX calls from the client to the server with JSON as the transport format.

#### Client

The web interface client part is a Rich Internet Application (RIA) managing the user interaction with the system. It has been written in Javascript and takes advantage of the object oriented nature of the language. The client uses the jQuery library to ease the DOM manipulation and to overcome the cross-browser compatibility issues. While offering a rich interactive user interface, easyDAS' UI needs were simple and so the interface is custom built and only minimal parts of the jQueryUI framework are used (i.e. dialog dragging functionality).

#### Server

The server side of the web interface has been implemented as a set of Perl CGI scripts running behind an Apache server. A custom easyDAS module provides the actual functionality and defines a basic file parser class. The different parsers are specializations of that class and completely independent of other parsers, so adding a new file parser to the system is quite straightforward and does not require the modification of any other file on the system.

### Database

The data back-end for easyDAS data is a MySQL relational database. The database is used to store both the information regarding the easyDAS system -such as which users are registered and what sources are available- and the actual biological data, the content of the DAS sources. The database schema was designed with the goal of isolating the sources as much as possible and so it has a set of six small tables for every source. This table setting maps very well to the hydra capability of the underlying DAS server and reduces the amount of code needed to implement the multiheaded DAS server. In addition, it simplyfies data insertion -no concurrent writes can happen on the same table- and deletion of sources -only whole table drops are required. Although the performance requirements of the database are low at the moment, it would be relatively easy to span the data over multiple database servers if it was needed.

This database is also accessible from the DAS server and so is the link between the two sides of the system.

### DAS Server

The DAS server in easyDAS is a ProServer with a minor customization. ProServer, written in Perl and already extensively used in the EBI systems, offers both the power and efficiency required to potentially serve hundreds or thousands of DAS sources.

One feature that sets ProServer apart from other DAS servers is its capability to create multi-headed DAS sources, what it calls a hydra source. Hydra sources do not require specific per-source configuration as any other source type, but can be created on the fly by a Perl function -which in easyDAS is querying the database- based on a single basic configuration. Without hydras, either the server must be restarted on every source modification or many individual servers should have been created, requiring much more server power than the single ProServer instance in use. While it is true that other approaches would have been possible, none of them is so simple and yet powerful as using ProServer hydras.

## Availability and requirements

• **Project name: **easyDAS

• **Project home page: **http://code.google.com/p/easydas/

• **Operating system(s): **Platform independent (web based)

• **Programming language: **Javascript, Perl

• **Other requirements: **none

• **License: **GNU LGPL

• **Any restrictions to use by non-academics: **none

## Authors' contributions

AJ, RJ and HH conceived the original idea of the system. BGM, AJ and RJ designed the system and BGM implemented it. AJ helped with the DAS server implementation. XMP and HH supervised the work. BGM drafted the manuscript. All authors read and approved the final manuscript.
